# Supplementary material for: The atypical chemokine receptor 3 interacts with Connexin 43 inhibiting astrocytic gap junctional intercellular communication
Source: Nat Commun. 2020 Sep 25;11:4855. doi: 10.1038/s41467-020-18634-y (PMC7519114; doi:10.1038/s41467-020-18634-y)
Supplement: Supplementary file 1 — Supplementary Information [file 41467_2020_18634_MOESM1_ESM.pdf]

The atypical chemokine receptor 3 interacts with Connexin 43 inhibiting astrocytic gap junctional intercellular communication

Fumagalli et al.

Protein	Host specie	Clone	Ref	Dilution & application	Supplier	Reactivity	Others	Lot number
**HA**	Rat	3F10	11867423001	1/1000 WB	Sigma-Aldrich	/		27573500
**HA**	Mouse	HA-7	A2095	50 uL/mg protein IP	Merck	/	Conjugated onto beads	057M4864V
**Cx30**	Rabbit	Z-PP9	71-2200	1/200 IF	Invitrogen	Mouse		UE288303
**Cx43**	Rabbit	Polyclonal	C6219	1/500 IHF<br>1/500 IF<br>1/5000 WB	Sigma-Aldrich	Human<br>Mouse		039M4814V
**Cx43**	Mouse	2/Connexin-43	610062	1/100 IF	BD	Human		4198911
**ACKR3**	Mouse	11G8	MAB42273	1/50 IF	R&D	Human	Antigen retrieval	YQU0214071
**CXCR4**	Rabbit	UMB2	ab124824	1/500 IF	Abcam	Human	Antigen retrieval	GR262216-6
**GFAP**	Mouse	Polyclonal	Z 0334	1/500 IHF	Dako	Mouse		20035993
**GFP**	Chicken	Polyclonal	A10262	1/1000 IHF	Invitrogen	/		2089131
**FLAG**	Mouse	M2	F1804	1/1000 WB	Sigma-Aldrich	/		088K6018

Supplementary Table 1 List of all primary antibody used

Figure	Test/Group	Time point	P Value
**Figure 1 d**	**Extra-sum-of Square, F-test (2,114)**		
	ACKR3		<0.0001
**Figure 1 e**	**Two-tailed unpaired t-test (2,2)**		
	ACKR3 *vs.*CXCR4		<0.0001
**Figure 1 f**	**Two-way Anova, Bonferroni (1,17)**		
	ACKR3 *vs.* CXCR4, R633		<0.0001
	ACKR3 *vs.* CXCR4, TG1		<0.0001
**Figure 1g**	**Two-way Anova, Bonferroni (22,99)**		
	CXCL12 *vs.* Vehicle	0:00:00,000	>0.9999
		0:02:54,030	>0.9999
		0:05:37,020	>0.9999
		0:08:33,050	0.0434
		0:11:15,000	0.0499
		0:13:57,000	0.0205
		0:16:51,020	0.0148
		0:19:41,010	0.0403
		0:22:14,040	0.0297
		0:25:03,030	0.0468
		0:27:52,010	0.0339
		0:30:41,000	0.0407
	CXCL11 *vs.* Vehicle	0:00:00,000	>0.9999
		0:02:54,030	>0.9999
		0:05:37,020	>0.9999
		0:08:33,050	0.9792
		0:11:15,000	0.302
		0:13:57,000	0.1675
		0:16:51,020	0.0663
		0:19:41,010	0.0469
		0:22:14,040	0.0221
		0:25:03,030	0.0094
		0:27:52,010	0.0111
		0:30:41,000	0.0072
**Figure 1h**	**One-way ANOVA, Bonferroni (2,12)**		
	CXCL12 vs. Vehicle		0.0003
	CXCL11 vs. Vehicle		0.0056
**Figure 2**	**One-way ANOVA, Bonferroni (11,24)**		
	CXCL12 *vs.* Vehicle		<0.0001
	CXCL11 *vs.* Vehicle		<0.0001
	CBX *vs.* Vehicle		<0.0001
	AMD3100 *vs.* Vehicle		>0.9999
	CXCL12 + AMD3100 *vs.* Vehicle		<0.0001
	NBI-74330 *vs.* Vehicle		>0.9999
	CXCL11 NBI-74330 *vs.* Vehicle		0.0004
	AMD3100 *vs.* AMD3100 + CXCL12		0.0002
	NBI-74330 *vs.* NBI-74330 + CXCL11		0.0016
	CXCL12 *vs.* AMD3100 + CXCL12		>0.9999
	CXCL11 *vs.* NBI-74330 + CXCL11		>0.9999
	**Two-way ANOVA, Bonferroni (3,16)**		
	Vehicle:Vehicle vs. PTX: Vehicle		0.0070
	Vehicle:Vehicle vs. PTX: CXCL11		0.0002
	Vehicle:Vehicle vs. PTX: CBX		<0.0001
	PTX:Vehicle vs. PTX: CXCL12		0.2990
	PTX:Vehicle vs. PTX: CXCL11		>0.9999
**Figure 3b**	**Two-way Anova, Bonferroni (87,573)**		
	Saline *vs.* CXCL12	0:00:30	>0.9999
		0:01:00	>0.9999
		0:01:30	>0.9999
		0:02:00	>0.9999
		0:02:30	>0.9999
		0:03:00	>0.9999
		0:03:30	>0.9999

		0:04:00	>0.9999
		0:04:30	>0.9999
		0:05:00	0.9186
		0:05:30	0.8542
		0:06:00	0.4227
		0:06:30	0.6943
		0:07:00	0.8138
		0:07:30	0.3355
		0:08:00	0.0717
		0:08:30	0.0194
		0:09:00	0.0003
		0:09:30	0.0013
		0:10:00	0.0014
		0:10:30	<0.0001
		0:11:00	0.0003
		0:11:30	0.0017
		0:12:00	0.0063
		0:12:30	0.0089
		0:13:00	0.0033
		0:13:30	0.0146
		0:14:00	0.0064
		0:14:30	0.0125
		0:15:00	0.0015
	Saline *vs.* CBX	0:00:30	>0.9999
		0:01:00	>0.9999
		0:01:30	>0.9999
		0:02:00	>0.9999
		0:02:30	>0.9999
		0:03:00	>0.9999
		0:03:30	>0.9999
		0:04:00	>0.9999
		0:04:30	>0.9999
		0:05:00	>0.9999
		0:05:30	>0.9999
		0:06:00	>0.9999
		0:06:30	>0.9999
		0:07:00	>0.9999
		0:07:30	>0.9999
		0:08:00	0.4698
		0:08:30	0.0091
		0:09:00	0.0037
		0:09:30	0.0064
		0:10:00	0.0013
		0:10:30	0.0006
		0:11:00	0.0002
		0:11:30	0.0002
		0:12:00	<0.0001
		0:12:30	<0.0001
		0:13:00	<0.0001
		0:13:30	0.0007
		0:14:00	<0.0001
		0:14:30	<0.0001
		0:15:00	0.0001
	Saline *vs.* CXCL11	0:00:30	>0.9999
		0:01:00	>0.9999
		0:01:30	>0.9999
		0:02:00	>0.9999
		0:02:30	>0.9999
		0:03:00	>0.9999
		0:03:30	0.8937
		0:04:00	0.5569
		0:04:30	0.4854

		0:05:00	0.1569
		0:05:30	0.1924
		0:06:00	0.1373
		0:06:30	0.14
		0:07:00	0.0159
		0:07:30	0.0192
		0:08:00	0.0155
		0:08:30	0.0093
		0:09:00	0.0005
		0:09:30	0.0028
		0:10:00	0.0053
		0:10:30	0.0001
		0:11:00	0.0031
		0:11:30	0.0048
		0:12:00	0.0003
		0:12:30	0.001
		0:13:00	0.0005
		0:13:30	0.0043
		0:14:00	0.0009
		0:14:30	0.0008
		0:15:00	0.0027
**Figure 3c**	**Two-tailed Wilcoxon test**		
	Saline Before *vs.* Saline after		0.6875
	CXCL12 Before *vs.* CXCL12 after		0.0313
	CXCL11 Before *vs.* CXCL11 after		0.0313
	CBX Before *vs.* CBX after		0.0313
**Figure 4a**	**Two-way Anova, Bonferroni (4,18)**		
	Vehicle:WT vs. Veichle:CXCL12		0.0068
	Vehicle:WT vs.Veichle:CXCL11		0.0026
**Figure 4b**	**Two-way Anova, Bonferroni (6,24)**		
	Vehicle:WT vs. CXCL12:WT		0.0108
	Vehicle:WT vs. CXCL11:WT		0.0072
	Vehicle:WT vs. CBX:WT		0.0006
	Vehicle:WT vs. CBX:DYN		<0.0001
	Vehicle:WT vs. CBX:KO		<0.0001
	CXCL12:WT vs. CXCL12:DYN		0.0076
	CXCL12:WT vs. CXCL12:KO		0.0180
	CXCL11:WT vs. CXCL11:DYN		0.0048
	CXCL11:WT vs. CXCL11:KO		0.0004
**Supplementary Figure 2**	**One-way ANOVA, Bonferroni(3,8)**		
	Vehicle *vs.* CXCL12		>0.9999
	Vehicle *vs.*CXCL11		>0.9999
	Vehicle *vs.*CBX		0.0009
**Supplementary Figure 3a**	**Unpaired two-way Anova, Bonferroni (3,16)**		
	Vehicle:fsk vs. Vehicle:fsk+CXCL12		<0.0001
	Vehicle:fsk vs. Vehicle:fsk+CXCL11		0.0254
	Vehicle:fsk vs. Vehicle:fsk+AMD3100+CXCL12		<0.0001
	Vehicle:CXCL12 vs. PTX: CXCL12		0.0020
	Vehicle:CXCL11 vs. PTX: CXCL11		<0,0001
	Vehicle:AMD+CXCL12 vs. PTX: AMD+ CXCL12		<0,0001
**Supplementary Figure 3b**	**Extra-sum-of Square, F-test (2,69)**		
	ACKR3		<0.0001
**Supplementary Figure 3c**	**Two-way Anova, Bonferroni (6,24)**		
	CXCR4:fsk vs. CXCR4:fsk+CXCL12		0.0018
	CXCR4:fsk+CXCL12+PTX vs. CXCR4:fsk+CXCL12		0.0054
**Supplementary Figure 4c**	**Extra-sum-of Square, F-test (2,49)**		
	ACKR3		<0.0001
	CXCR4		0.098
**Supplementary Figure 4e**	**Two-way Anova, Bonferroni (2,12)**		
	Cx43:Vehicle vs. Cx43:CXCL12 30min		0.0328
	B-arr2:Vehicle vs. B-arr2:CXCL12 30min		0.0155

Supplementary Table 2. Statistical test and exact P-values for each Figure are here reported.

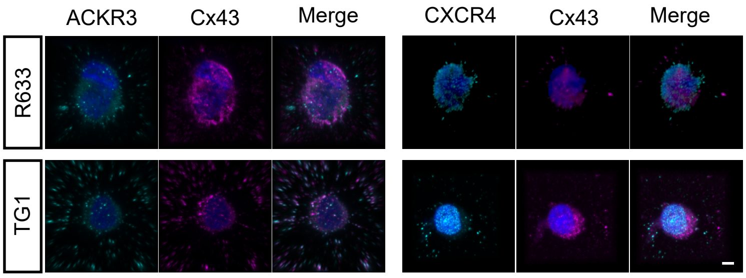

Supplementary Figure 1. Staining of R633 and TG1 glioma cells for Cx43 and either ACKR3 or CXCR4. Original confocal images corresponding to the 3D reconstruction of immunoreactive signals illustrated on Figure 1f are illustrated (measured in 5 (ACKR3, TG1 and R633), 4 (CXCR4, TG1) or 7 (CXCR4, R633) cells). Scale bar = 3mm.

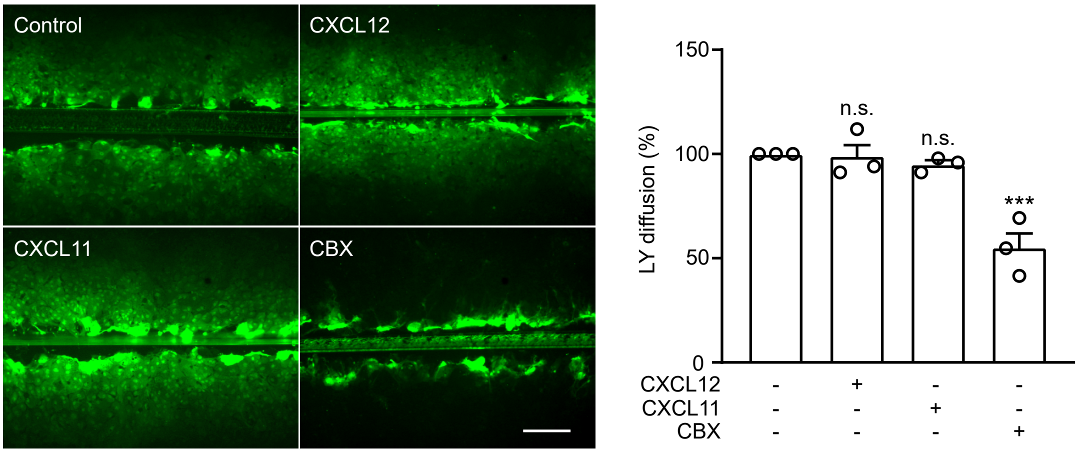

Supplementary Figure 2. 5-min ACKR3 activation does not inhibit GJIC. Representative photomicrographs of scrape loading performed in confluent primary astrocytes exposed to CXCL12 (10 nM) or CXCL11 (100 nM) for 5 min. CBX (50 μM) was applied overnight. Scale bar = 200 μm. LY diffusion was calculated by measuring the distance from the scrape where LY fluorescence intensity is 50% of the maximal fluorescence. Values were normalized to LY diffusion in vehicle-treated astrocytes. Results represent the means ± SEM of values obtained from three independent experiments performed in duplicate from different sets of cultured cells (One-way Anova, Bonferroni post-hoc, F (3,8)). See the Statistics and Reproducibility section for exact P values and symbol (*) legend. Source data and are provided as a Source Data file.

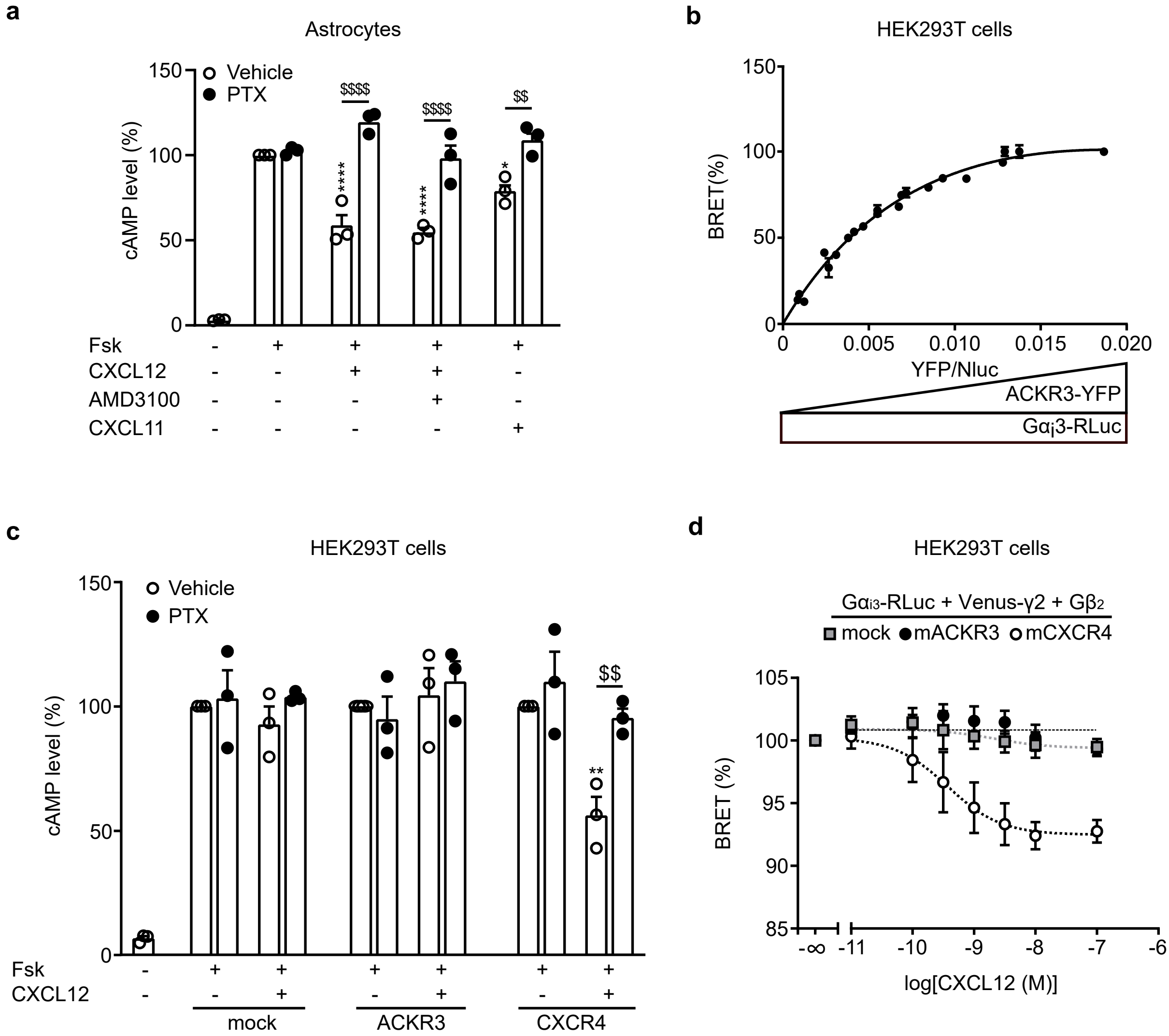

Supplementary Figure 3. Cell-type dependent involvement of G proteins in ACKR3-mediated effects. (a) Quantification of cAMP production in primary astrocyte cultures treated for 5 min with vehicle or the adenylyl cyclase activator forskolin (FSK, 1 μM) in absence or presence of either CXCL12 (10 nM) or CXCL12 + AMD3100 (10 μM, added for 30 min before the CXCL12 challenge) or CXCL11 (100 nM) for 5 min. Values are the means ± SEM results obtained in three independent experiments (each performed in triplicate on different sets of cultured cells) (Two-way ANOVA, Bonferroni post-hoc, F (3,16)). (b) Saturation curves of ACKR3-YFP/Gαi3-RLuc interaction in HEK293T cells. BRET values were normalized to the maximum BRET measured in each replicate. They are the means ± SEM of results obtained in three biological replicates. The one site total curve was fitted as described in the legend to Figure 1d (Extra-sum-of Square, F-test, $p < 0.0001$, F (2,69)). (c) cAMP production in HEK293T cells transiently transfected with empty plasmid (Mock) or cDNAs encoding ACKR3 or CXCR4 treated or not with PTX (100 ng/mL, 18 h) and exposed to forskolin (Fsk, 1 μM) for 5min in absence or presence CXCL12 (10 nM). Data are the means ± SEM of values obtained in three independent experiments, each performed in triplicate (Two-way ANOVA, Bonferroni post-hoc, F (6,24)). (d) HEK293T cells transiently expressing RLluc-Gαi3 protein, Venus-γ2 and β2 alone (mock) or in combination with mouse ACKR3 (mACKR3) or mouse CXCR4 (mCXCR4) were challenged for 5 min with incremental concentrations of CXCL12. Data represent the mean ± SEM of normalized BRET values measured in three independent biological replicates. See the Statistics and Reproducibility section for the exact P values and symbol (*,$) legend. Source data are provided as a Source Data file.

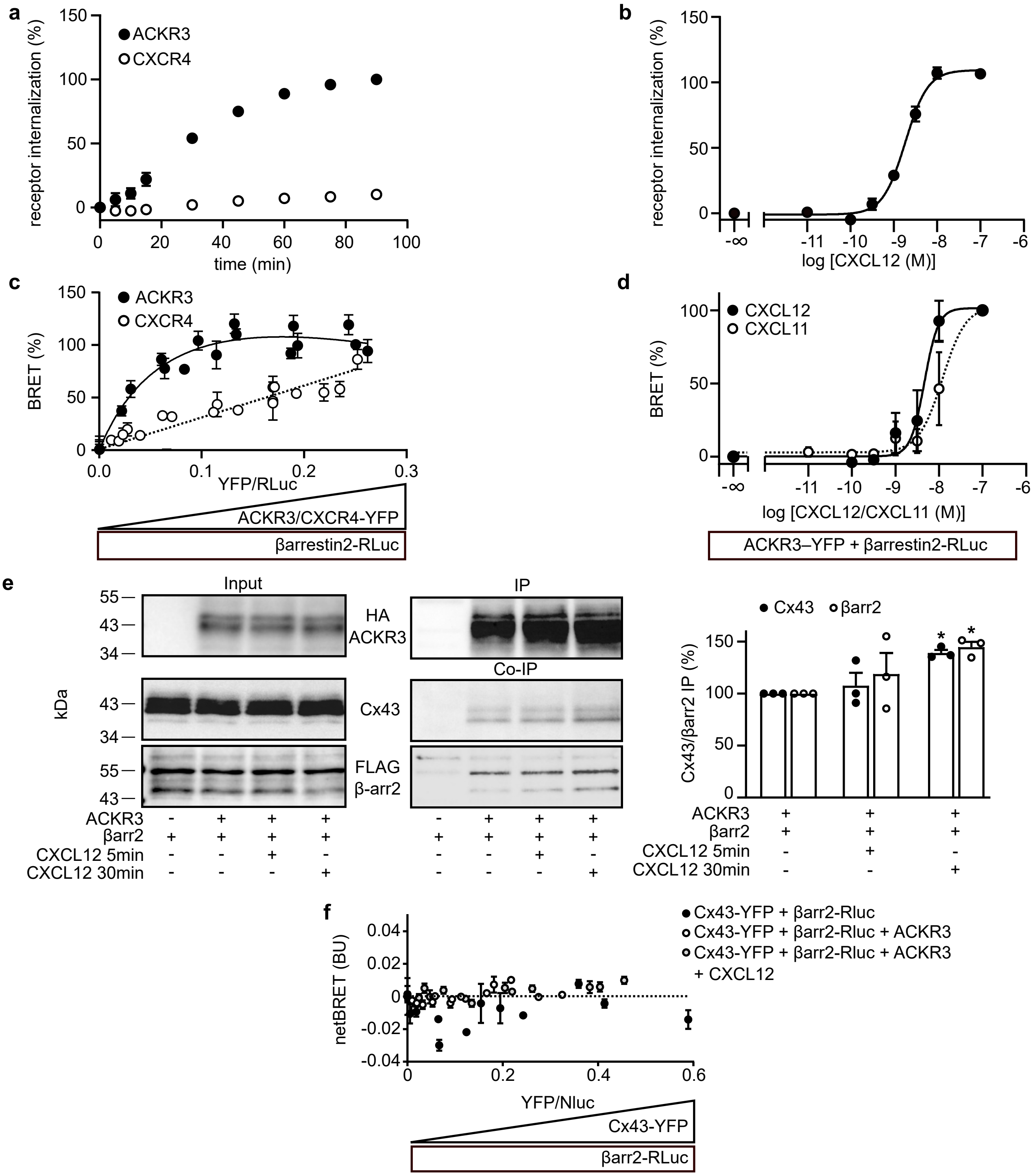

Supplementary Figure 4. ACKR3 recruits β-arrestin2. (a) Constitutive internalization of SNAP-tagged ACKR3 and CXCR4 transiently expressed in HEK293T cells, as assessed by using the DERET assay. Data represent the means ± SEM of values (normalized to the maximal internalization) obtained in three biological replicates. (b) SNAP-tagged ACKR3 internalization in HEK293T cells exposed for 45 min to incremental CXCL12 concentrations. Data represent the means ± SEM of values (normalized to ACKR3 internalization elicited by the highest CXCL12 concentration) obtained in three independent replicates (each performed in triplicate). (c) Quantification of the BRET signal between β-arrestin2-Rluc and either ACKR3-YFP or CXCR4-YFP expressed in HEK293T cells. Results represent means ± SEM of BRET values (normalized to the maximum BRET obtained in each replicate) obtained in three independent experiments (each performed in triplicate). The One-site total curve and line through the origin were fitted as described in the legend to Figure 1d. (Extra-sum-of Square, F-test, $F_{(2,49)}$). (d) Quantification of BRET signals between YFP-ACKR3 and β-arrestin2-Rluc in HEK293T cells exposed to incremental concentrations of CXCL12 or CXCL11 for 5 min. Data represents the means ± SEM of values (normalized to the maximum BRET obtained in each replicate) obtained in three independent replicates (each performed in triplicate). (e) Representative Western blots of HA-immunoprecipitations in HEK293T cells transiently expressing HA-ACKR3 and Flag-β-arrestin2 and exposed or not to CXCL12 (10 nM) for 5 or 30 min. The histogram shows the means ± SEM of immunoreactive signals (normalized to the values obtained in cells not challenged with CXCL12) of Cx43 and Flag-β-arrestin2 co-immunoprecipitated with HA-ACKR3 in three independent experiments (Two-way ANOVA, Bonferroni post-hoc $F_{(2,12)}$). (f) Saturation curves of Cx43-YFP/β-arrestin2-Rluc interaction in HEK293T cells in presence or absence of HA-ACKR3 and exposed or not to CXCL12 (10 nM). They are the means ± SEM of results obtained in three biological replicates. See the Statistics and Reproducibility section for the exact P values and symbol (*) legend. Source data and uncropped WB are provided as a Source Data file.

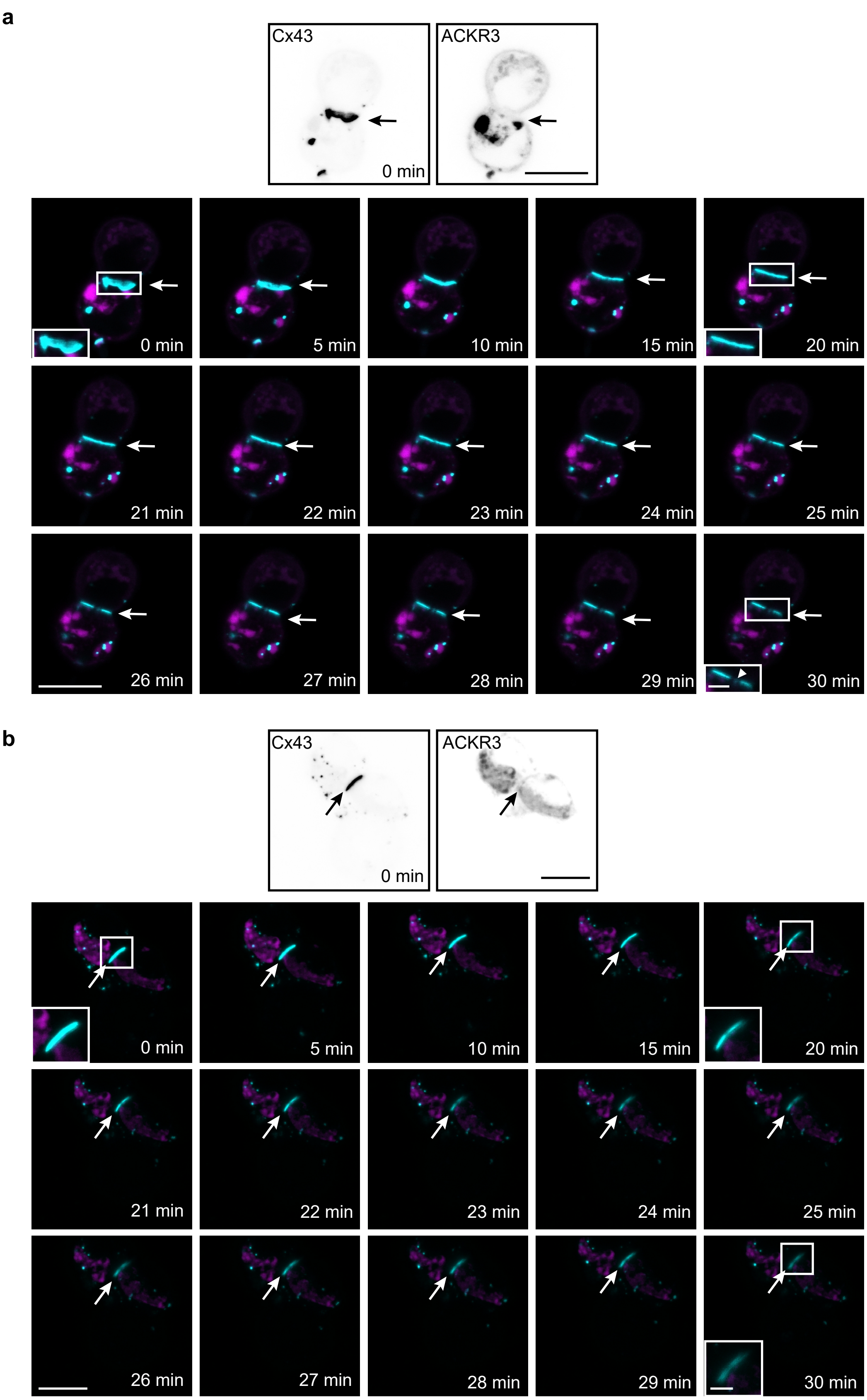

Supplementary Figure 5. ACKR3 activation promotes Cx43 internalization. (a,b) Time-lapse images of gap junction plaques in HEK293T cells co-expressing Cx43-GFP and RedCherry-ACKR3 and exposed to CXCL12 (10 nM) (a) or CXCL11 (100 nM) (b) for 30 min. The two upper images for each panel show GFP (Cx43, left panel) and RedCherry (ACKR3, right panel) fluorescent signals, respectively, before the onset of chemokine application. Images below show the gap junction plaques at the indicated times. A zoomed image of a Cx43-GFP plaque is represented at 0, 20 and 30 min in the left bottom corner of the image. The arrowhead shows the removal of the center part of the plaque. The experiments was conducted in three biologically independent replicates with similar results. Scale bar = 10 and 2.5 μm for the time lapse and magnification, respectively.

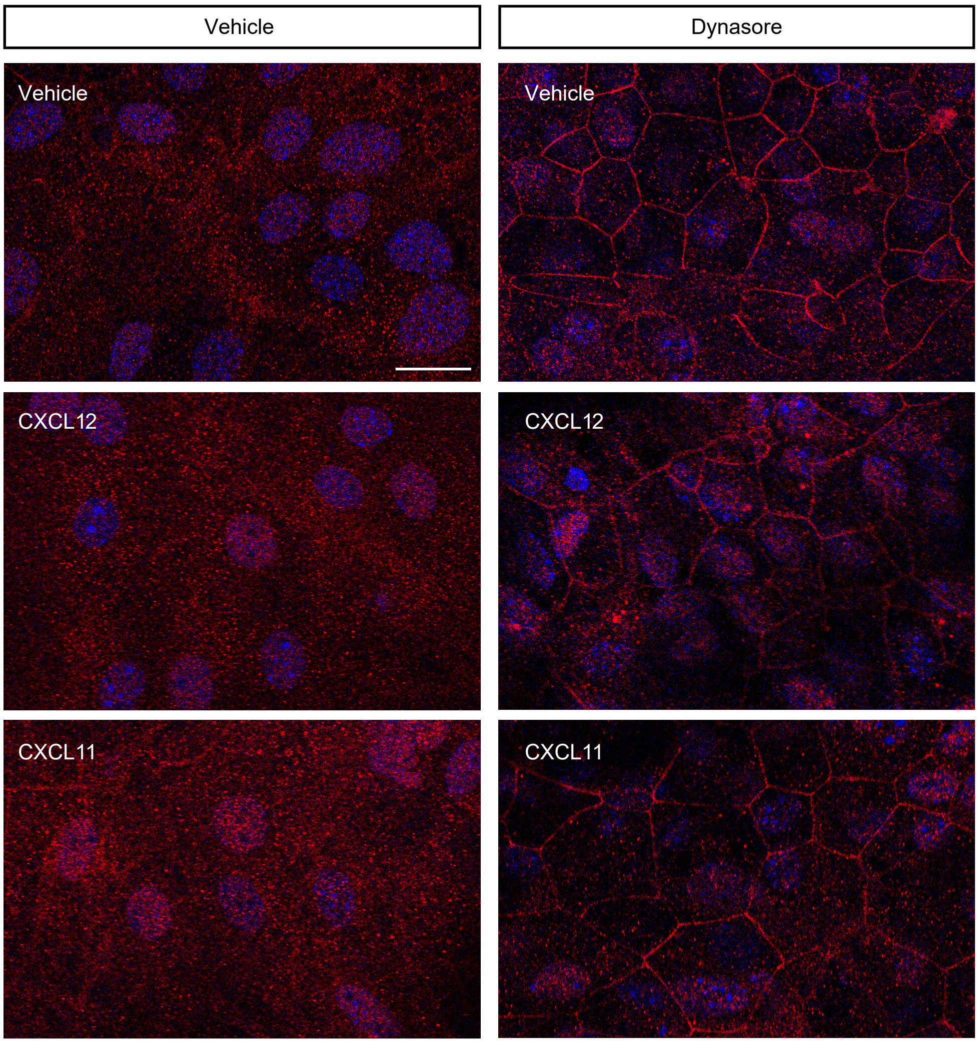

Supplementary Figure 6. ACKR3 stimulation does not affect Cx30 cellular localization. Confocal images of Cx30 immunostaining in confluent primary astrocyte cultures exposed for 30 min to either vehicle or CXCL12 (10 nM) or CXCL11 (100 nM) in the absence or presence of Dynasore (80 mM). Scale bar = 25 µm. Representative images of three independent experiments performed on three different sets of cultured cells, wit similar results, are illustrated.
