# Supplementary material for: The atypical chemokine receptor 3 interacts with Connexin 43 inhibiting astrocytic gap junctional intercellular communication
Source: Nat Commun. 2020 Sep 25;11:4855. doi: 10.1038/s41467-020-18634-y (PMC7519114; doi:10.1038/s41467-020-18634-y)
Supplement: Supplementary file 3 — Description of Additional Supplementary Files [file 41467_2020_18634_MOESM3_ESM.pdf]

Description of Additional Supplementary Files

File Name: Supplementary Data 1

Description: **List of proteins that specifically co-immunoprecipitate with ACKR3 in HEK293T cells.** Proteins statistically enriched according to their LFQ value in the ACKR3 complex, compared to Mock cells, are reported. Protein name, Uniprot ID, gene name, log2 of the LFQ difference between ACKR3 and mock cell (Difference) and the – log P values are indicated. The statistical analysis was performed using the Perseus software as detailed in the "Statistics and Reproducibility" section. Proteins are ranked based on their difference in abundance in immunoprecipitates from ACKR3-expressing cells vs. Mock cells. The bait ACKR3 is shown in blue, canonical GPCR interacting proteins in light blue and the accessory proteins of Rab and Ubiquitin complexes as well as MAP2K2 are depicted in green. GJA1 is highlighted in red and the proteins already known to interact with it (at least functionally) in orange.
