# Supplementary material for: The atypical chemokine receptor 3 interacts with Connexin 43 inhibiting astrocytic gap junctional intercellular communication
Source: Nat Commun. 2020 Sep 25;11:4855. doi: 10.1038/s41467-020-18634-y (PMC7519114; doi:10.1038/s41467-020-18634-y)
Supplement: Supplementary file 5 — Reporting Summary [file 41467_2020_18634_MOESM5_ESM.pdf]

nature research

Corresponding author(s): Philippe MARIN

Last updated by author(s): Jul 3, 2020

Reporting Summary

Nature Research wishes to improve the reproducibility of the work that we publish. This form provides structure for consistency and transparency in reporting. For further information on Nature Research policies, see our Editorial Policies and the Editorial Policy Checklist.

Statistics

For all statistical analyses, confirm that the following items are present in the figure legend, table legend, main text, or Methods section.

n/a	Confirmed	
☐	☒	The exact sample size (*n*) for each experimental group/condition, given as a discrete number and unit of measurement
☐	☒	A statement on whether measurements were taken from distinct samples or whether the same sample was measured repeatedly
☐	☒	The statistical test(s) used AND whether they are one- or two-sided<br>*Only common tests should be described solely by name; describe more complex techniques in the Methods section.*
☒	☐	A description of all covariates tested
☒	☐	A description of any assumptions or corrections, such as tests of normality and adjustment for multiple comparisons
☐	☒	A full description of the statistical parameters including central tendency (e.g. means) or other basic estimates (e.g. regression coefficient) AND variation (e.g. standard deviation) or associated estimates of uncertainty (e.g. confidence intervals)
☐	☒	For null hypothesis testing, the test statistic (e.g. F, t, r) with confidence intervals, effect sizes, degrees of freedom and P value noted<br>*Give P values as exact values whenever suitable.*
☒	☐	For Bayesian analysis, information on the choice of priors and Markov chain Monte Carlo settings
☒	☐	For hierarchical and complex designs, identification of the appropriate level for tests and full reporting of outcomes
☒	☐	Estimates of effect sizes (e.g. Cohen's d, Pearson's r), indicating how they were calculated

Our web collection on statistics for biologists contains articles on many of the points above.

Software and code

Policy information about availability of computer code

Data collection	XCalibur software (v 2 .2), Axiovision 4.8 software (Zeiss), MicroWin200 software (Berthold Biotechnologies), LAS-X (v3.5, Leica).
Data analysis	MaxQuant (v 1.5.5.1) including the LFQ algorithm Perseus (v 1.5.6.072), Fiji (v2.0.0), Imaris software (Bitplane, v 7.2), Prism 8 (GraphPad software), Image Lab (Bio-Rad), LAS-X (v3.5, Leica).

For manuscripts utilizing custom algorithms or software that are central to the research but not yet described in published literature, software must be made available to editors and reviewers. We strongly encourage code deposition in a community repository (e.g. GitHub). See the Nature Research guidelines for submitting code & software for further information.

Data

Policy information about availability of data

All manuscripts must include a data availability statement. This statement should provide the following information, where applicable:

- Accession codes, unique identifiers, or web links for publicly available datasets
- A list of figures that have associated raw data
- A description of any restrictions on data availability

The source data underlying all the data together with the uncropped version of the blots are provided as a Source Data File (Source Data File). Mass spectrometry data have been deposited to the ProteomeXchange Consortium via the PRIDE partner repository with the dataset identifier PXD016978.

Field-specific reporting

Please select the one below that is the best fit for your research. If you are not sure, read the appropriate sections before making your selection.

- [x] Life sciences
- [ ] Behavioural & social sciences
- [ ] Ecological, evolutionary & environmental sciences

For a reference copy of the document with all sections, see nature.com/documents/nr-reporting-summary-flat.pdf

Life sciences study design

All studies must disclose on these points even when the disclosure is negative.

Sample size	Three biological replicates (performed on different sets of cultured cells) were performed for the initial interactomic screen, according to the guidelines of high-ranked proteomics journals such as Mol. Cell Proteomics (see Schmoker et al. Mol. Cell Proteomics, 2020). In further studies performed to functionally validate the interaction between ACKR3 and Cx43, sample sizes and number of replicates were chosen according to previous publications in the field (see Meyrath et al. Nature Communication,2020), so as to allow to demonstrate reproducibility of the measurements and generate statistically significant results. A minimum of three biological replicates (independent sets of cultured cells or different animals) was performed for each experiment.
Data exclusions	No data were excluded from the analyses.
Replication	Number of independent experiments and replicates are in the Statistics and Reproducibility section. All experiments could be reliably reproduced with little variability between the experiments (see S.E.M for the respective experiments).
Randomization	No particular randomization was used. Most of the measurements were performed without human intervention using pre-established methods on automatic instruments.
Blinding	Since each experiment was perfomed by a single investigator, it was not possible to be blind during data collection and analysis. Furthermore, the data collected are quantitative and not subjected to investigator bias.

Reporting for specific materials, systems and methods

We require information from authors about some types of materials, experimental systems and methods used in many studies. Here, indicate whether each material, system or method listed is relevant to your study. If you are not sure if a list item applies to your research, read the appropriate section before selecting a response.

Materials & experimental systems

n/a	Involved in the study	
[ ]	[x]	Antibodies
[ ]	[x]	Eukaryotic cell lines
[x]	[ ]	Palaeontology and archaeology
[ ]	[x]	Animals and other organisms
[x]	[ ]	Human research participants
[x]	[ ]	Clinical data
[x]	[ ]	Dual use research of concern

Methods

n/a	Involved in the study	
[x]	[ ]	ChIP-seq
[x]	[ ]	Flow cytometry
[x]	[ ]	MRI-based neuroimaging

Antibodies

Antibodies used

anti HA produced in Rat Clone 3F10 Sigma Ref. 11867423001 lot number 27573500
anti-HA produced in Mouse clone HA-7 beads conjugated Merck A2095 lot number 057M4864V
anti Connexin43 produced in Rabbit Polycolonal C6219 Sigma Ref. C6219 lot number 039M4814V
anti Connexin43 produced in Mouse Clone 610062 BD Ref. 610062 lot number 4198911
anti ACKR3 produced in Mouse Clone 11G8 R&D Ref. MAB42273 lot number YQU0214071
anti CXCR4 produced in Rabbit Clone UMB2 Abcam Ref. ab124824 lot number GR262216-6
anti GFAP produced in Mouse Polycolonal Dako Ref. Z0334 lot number 20035993
anti GFP produced in Chicken Polyclonal Invitrogen Ref. A10262 lot number 2089131
anti Cx30 produced in Mouse clone Z-PP9 Invitrogen Ref 71-2200 lot number UE288303
anti FLAG produced in Mouse clone Sigma-Aldrich M2 F1804 lot number 088K6018
anti mouse conjugated to HRP Merck Ref. GENA931V lot number 2470357
anti rat conjugated to HRP Jackson ImmunoResearch Ref. 112-035-003 lot number 116996
anti rabbit conjugated to HRP Merck Ref. GENA934V lot number 0704057822
anti chicken Alexa Fluor 488 Thermo Fisher Scientific Ref. A-11039 lot number 745484
anti rabbit Alexa Fluor 594 Thermo Fisher Scientific Ref. A-11037 lot number 483570
anti mouse Alexa Fluor 680 Thermo Fisher Scientific Ref. A-21057 lot number 1214851

	anti mouse Alexa Fluor 488 Thermo Fisher Scientific Ref. A-11029 lot number 235368
Validation	The anti HA rat antibody is routinely used in our laboratory and has been found specific comparing HEK293T cells transiently expressing the proteins of interested. This can be observed in multiple WB present in the paper, such as Figure 1e.<br>The anti-HA mouse is routinely used in a large body of publications (more than 320 publications as indicated in the manufacturer's web site,(https://www.sigmaaldrich.com/catalog/product/sigma/a2095?lang=it&region=IT)<br>The ACKR3 clone 11G8 was found specific in the following publication (doi:10.1016/j.imlet.2010.06.010) and used in at least other 18 publications as indicated in the manufacturer's web site (https://www.rndsystems.com/products/human-cxcr7-rdc-1-antibody-11g8_mab42273).<br>The UMB2 CXCR4 antibody was characterized in the following publication (https://doi.org/10.1371/journal.pone.0004069) and used in at least other 87 publications as indicated in the manufacturer's web site (https://www.abcam.com/cxcr4-antibody-umb2-ab124824-references.html#active-tab).<br>The polycolonal Cx43 antibody is routinely used in a large number of publications. As indicated in the manufacturer's web site (https://www.sigmaaldrich.com/catalog/product/sigma/c6219?lang=fr&region=FR), it was used in more than 250 publications.<br>The mouse Cx43 antibody was used in at least five publications as indicated in the manufacturer's web site (http://www.bdbiosciences.com/eu/applications/research/stem-cell-research/mesoderm-markers/mouse/purified-mouse-anticonnexin-43-2connexin-43/p/610062).<br>The GFP antibody is routinely used in a large body of publications (more than 130 publications as indicated in the manufacturer's web site, https://www.thermofisher.com/antibody/product/GFP-Tag-Antibody-Polyclonal/A10262).<br>The GFAP antibody is routinely used in a large body of publications (more than 1,000 publications, as indicated in the manufacturer's website: https://www.citeab.com/antibodies/2452274-z0334-glial-fibrillary-acidic-protein-gfap) .<br>The Cx30 antibody s routinely used in a large body of publications (more than 100 publications as indicated in the manufacturer's web site, https://www.thermofisher.com/antibody/product/Connexin-30-Antibody-clone-Z-PP9-Polyclonal/71-2200).<br>The FLAG antibody is routinely used in a large body of publications (more than 4000 publications as indicated in the manufacturer's web site, https://www.sigmaaldrich.com/catalog/product/sigma/f1804?lang=it&region=IT)

Eukaryotic cell lines

Policy information about cell lines

Cell line source(s)	1)HEK293T cells directly purchased from the American Type Culture Collection (Anassas, VI, ATCC, CRL-3216™).<br>2)TG1 and R633 cell lines described in references 60 and 61 of the manuscript. Source Neuroscience Paris Seine-IBPS, Sorbonne Universities, 75005, Paris, France.
Authentication	1) Not further authenticated.<br>2) Characterized for their stem-like and tomour initiating properties. Authentication of human glioblastoma cell lines is routinely performed by short tandem repeat (STR) assays since their initial isolation.
Mycoplasma contamination	Absence of mycoplasma was assessed every month using the MycoAlert Mycoplasma Detection Kit (Lonza, Ref LT07-118) as indicated in the Methods section.
Commonly misidentified lines<br>(See ICLAC register)	No commonly misidentified lines were used in this study.

Animals and other organisms

Policy information about studies involving animals; ARRIVE guidelines recommended for reporting animal research

Laboratory animals	CD-1 EGFP-ACKR3 BAC mice (6-week old) were subjected to IHC without considering their sex.<br>Embryonic WT or C57BL/6J beta-arrestin 2 KO mice (E16.5) were used without determining their sex.<br>Embryonic WT or C57BL/6 HA-ACKR3 mice (E17) were used without determining their sex.<br><br>Mice were housed under standardized conditions with a 12-h light/dark cycle, stable temperature (22 ± 1°C), controlled humidity (55 ± 10%), and free access to food and water.
Wild animals	This study did not involve wild animals.
Field-collected samples	This study did not involve samples collected from the field.
Ethics oversight	Experiments on animals conformed to European ethics standards (86/609-EEC) and to decrees of the French National Ethics Committee (N°87/848) for the care and use of laboratory animals. Protocols were approved by regional ethic committee for animal use (CEEA LR 34, #7251).

Note that full information on the approval of the study protocol must also be provided in the manuscript.
